# Evaluation of a Commercial Ozonated Olive Oil Product (800 mEq O_2_/Kg) Against Methicillin-Resistant *Staphylococcus pseudintermedius* (MRSP) Using an Ex Vivo Canine Skin Model

**DOI:** 10.3390/pathogens15030323

**Published:** 2026-03-18

**Authors:** Hilke Oltmanns, Aimara Bello Suarez-Kupka, Christina Puff, Jessica Meißner, Andrea Vanessa Volk

**Affiliations:** 1 Department of Pharmacology, Toxicology and Pharmacy, University of Veterinary Medicine Hannover Foundation, 30559 Hannover, Germany; hilke.oltmanns@tiho-hannover.de (H.O.); jessica.meissner@tiho-hannover.de (J.M.); 2 Hospital for Small Animals, University of Veterinary Medicine Hannover Foundation, 30559 Hannover, Germany; andrea.volk@tiho-hannover.de; 3 Department of Pathology, University of Veterinary Medicine Hannover Foundation, 30559 Hannover, Germany; christina.puff@tiho-hannover.de

**Keywords:** ozonated oil, MRSP, ex vivo skin model, Franz diffusion cell, canine, antibacterial therapy

## Abstract

Background: Methicillin-resistant *Staphylococcus pseudintermedius* (MRSP) represents an emerging challenge in veterinary dermatology. Commercially available ozonated oils promise antibacterial activity, but their efficacy under physiologically relevant conditions remains unexplored. Objective: We aimed to evaluate the efficacy of commercial ozonated olive oil product (800 mEq O_2_/kg) against MRSP using an established in vitro model and a newly presented ex vivo canine skin model. Materials and Methods: In vitro susceptibility testing determined minimum inhibitory concentrations (MIC) and time–kill kinetics. Subsequently, canine skin samples were mounted in Franz diffusion cells, inoculated with MRSP (~10^6^ colony-forming units [CFU]), and treated for 8 h with ozonated or placebo olive oil. Bacterial viability was assessed by quantitative culture and histopathology. Results: In vitro testing demonstrated antibacterial activity for ozonated oil (MIC < 20% *v*/*v*) compared to placebo oil (90% *v*/*v*), with ozonation-specific bactericidal effects. However, ex vivo testing showed no MRSP reduction for either oil versus untreated controls, with bacterial localization in superficial dermis unchanged. Conclusions: Despite in vitro activity, this ozonated olive oil failed to reduce MRSP in ex vivo skin, revealing that tissue barriers prevent antibacterial delivery. These findings demonstrate that in vitro screening cannot predict topical efficacy and emphasize the necessity of tissue-based validation before clinical use.

## 1. Introduction

*Staphylococcus* (S.) *pseudintermedius* is a Gram-positive, coagulase-positive coccus and the primary causative agent of bacterial skin infections in dogs, including superficial and deep pyoderma. Over the past two decades, the emergence of methicillin-resistant *S. pseudintermedius* (MRSP) has become a major therapeutic concern in veterinary dermatology and a zoonotic risk for humans. The European Food Safety Authority recognizes *S. pseudintermedius* among the most relevant antibacterial-resistant bacteria in companion animals, with prevalence rates approaching 30% in clinical isolates [1]. MRSPs’ ability to form biofilms and acquire multidrug resistance genes severely limits the effectiveness of conventional topical and systemic antibiotics, prompting the need for novel non-antibiotic strategies [2].

Ozone (O_3_), a triatomic form of oxygen, exhibits broad-spectrum antibacterial, anti-inflammatory, and wound-healing properties [3]. In detail, it oxidizes bacterial cell-wall phospholipids and lipoproteins, disrupts membrane integrity, and impairs enzymatic functions essential for bacterial survival. It has also been demonstrated in vitro that there is an ability to disrupt biofilms and to stimulate local antioxidant and immune responses, thereby enhancing tissue repair [4,5].

Plant-derived triglyceride oils such as olive (*Olea europaea*) and sunflower (*Helianthus annuus*) oils are widely used as topical emollients and vehicles in human and veterinary dermatology and serve as stable carriers that release reactive oxygen species gradually, allowing safe topical use [6,7]. Olive oil and sunflower oil are closely related in chemical class and function, making them the most used base oils for ozonation [6,8].

Several veterinary studies have reported accelerated healing of cutaneous wounds in dogs and cats treated with ozonated vegetable oils, achieving faster recovery compared with standard protocols [5,9]. More recently, standardized ozonated oils produced with medical-grade generators have shown dose-dependent (mEq O_2_/kg) bactericidal effects against *Staphylococcus aureus, Enterococcus faecalis, Escherichia coli*, and *S. pseudintermedius* isolates of veterinary origin [10].

Currently, several commercial products claim antibacterial activity associated with the product’s mEq/O_2_/kg; however, data on the effect of single commercially available ozonated oils against MRSP in canine skin remain scarce, and no controlled ex vivo infection models have yet been used to evaluate their antibacterial efficacy. Ex vivo skin models offer a valuable intermediate step between in vitro susceptibility testing and in vivo studies, providing a tissue-based environment that more closely approximates clinical conditions while adhering to the 3R principles (Replace, Reduce, Refine) of ethical animal research. Such models allow preliminary efficacy screening of novel antibacterial candidates before proceeding to in vivo trials, thereby reducing unnecessary animal use. Building on recent advances in ex vivo canine skin systems developed for MRSP and phage studies [2], we aimed to evaluate the efficacy of a commercial ozonated olive oil product (800 mEq O_2_/kg) against MRSP using an established in vitro model and a newly presented ex vivo canine skin model.

## 2. Materials and Methods

### 2.1. Antibacterial Testing In Vitro

For the microdilution assay, *S. pseudintermedius* (strain 5463/1/22) was grown on Columbia sheep blood agar (Oxoid, Wesel, Germany) for 18 to 20 h and suspended in 0.9% saline solution at an optical density of 0.5 McFarland (approximately 1.5 × 10^8^ CFU/mL). The tested oils were dissolved and diluted in cation-adjusted Müller–Hinton broth (Sigma-Aldrich, Steinheim, Germany) at concentrations of 20% to 90% for ozonated oil and placebo oil. This concentration range was selected to encompass the full spectrum of potential antibacterial activity, with 20% representing the lowest practically relevant concentration and 90% representing the maximum achievable concentration in aqueous broth while maintaining bacterial viability. Concentrations were tested in 10% increments (20%, 30%, 40%, 50%, 60%, 70%, 80%, 90%) to allow precise determination of the MIC threshold. On a U-bottom 96-well plate (Greiner, Frickhausen, Germany), 180 µL of medium with oil was mixed with 20 µL of bacterial suspension. The positive control included only broth with bacterial suspension, and the negative control included only 20 µL saline solution. An additional negative control contained only ozone oil or placebo and saline. After incubation for 20 to 24 h, the minimal inhibitory concentration (MIC) was analyzed visually. According to the Clinical and Laboratory Standards Institute (CLSI), the MIC is defined as the lowest concentration of tested substances that shows no bacterial growth. The assay was repeated five times in biological replicates.

The time–kill kinetic analyses, assessing bactericidal activity over defined contact periods, were performed according to Petani et al. [11], with modification. *S. pseudintermedius* was grown on Columbia sheep blood agar for 18 to 20 h and suspended in 0.9% saline solution at an optical density of 0.5 McFarland, approximately 1.5 × 10^8^ colony-forming units (CFU/mL) [12]. In total, 900 µL ozonated oil or placebo oil was mixed with 100 µL bacterial suspension (containing approximately 1.5 × 10^7^ CFU, i.e., 15 million CFU) and vortexed for 10 s. The tubes were incubated for 5, 10, 20, and 60 min at 37 °C. Then, 100 µL of the mixture was plated on Columbia sheep blood agar, in duplicate, and incubated for 24 h at 37 °C before CFU values were counted. The time–kill kinetic assay was repeated six times in biological replicates.

### 2.2. Skin Samples

Skin samples were collected from euthanized dogs at the Small Animal Hospital, University of Veterinary Medicine Hannover Foundation. Euthanasia was performed for medical reasons unrelated to this study. The skin was kindly donated for research purposes by the owners. Five dogs were included, representing short-coated breeds with predominantly telogen hair cycles; please see Appendix A, Table A3, for complete donor characteristics. All skin donor sites were macroscopically inspected at the time of collection by a veterinarian for any signs of dermatological disease (e.g., erythema, pustules, crusting, alopecia, scaling). Dogs with clinically altered skin at the collection site were excluded, as were dogs that had received systemic antibacterial therapy within 72 h prior to euthanasia. One dog had been treated with trimethoprim-sulfadimidine; however, the MRSP strain used in this study demonstrated resistance to this antibiotic according to antibacterial susceptibility testing (see Appendix A, Table A1).

Skin samples were collected from the lateral thoracic region within 4 h of euthanasia following the protocol previously reported by Ehling et al. [2]. This region was selected because (1) it provides a relatively large, flat area of uniform skin thickness suitable for obtaining multiple standardized 3 × 3 cm samples; (2) this region is a common site of superficial pyoderma in dogs, making it clinically relevant; (3) the lateral thorax has consistent skin architecture with minimal regional variation in hair follicle density and dermal thickness compared to other body regions; and (4) this collection site is consistent with the validated protocol established by Ehling et al. [2], enabling direct comparability between studies. Hair was clipped in a 20 × 20 cm section, and the skin was excised. The subcutis was carefully removed using a scalpel blade, and the remaining tissue was stored at −20 °C for a maximum of 6 months. According to the Organisation for Economic Co-operation and Development (OECD) guidelines, frozen skin maintains structural and barrier integrity for up to 466 days when stored at −20 °C [12].

### 2.3. Methicillin-Resistant Staphylococcus pseudintermedius (MRSP)

The MRSP isolate used in this study (strain 5463/1/22) was obtained from Ehling et al. [2], where it was originally isolated from a wound of a dog at the Hospital for Small Animals, University of Veterinary Medicine Hannover Foundation. The strain has been comprehensively characterized and validated for use in this ex vivo model system [2]. The MIC values were determined according to the CLSI guideline VET01, 5th edition [13], and oxacillin resistance was confirmed by both phenotypic testing and PCR detection of the mecA gene [2]. The MIC of fusidic acid against this specific MRSP strain was previously determined by Ehling et al. [2], confirming its use as a positive antibacterial control (see Appendix A, Table A1).

### 2.4. Ozonated Olive Oil and Control Formulations

Detailed chemical composition data beyond peroxide value were proprietary and not fully disclosed by the manufacturer. The ozonated olive oil derived from *Olea europaea* was characterized only by its peroxide value (800 mEq O_2_/kg). The pre-ozonation base olive oil, obtained from the same manufacturer to ensure identical fatty acid composition, with ozonation as the only experimental variable, represented standard food-grade olive oil composition. No additional additives, preservatives, emulsifiers, or excipients were present in either formulation according to manufacturer certification.

Each treatment consisted of 300 µL of oil applied topically to a 3 × 3 cm skin sample (9 cm^2^ surface area), corresponding to approximately 33 µL/cm^2^. The oil was applied evenly across the skin surface and gently massaged into the tissue for uniform distribution.

#### Experimental Controls

Positive controls consisted of skin samples inoculated with MRSP without any treatment intervention, representing untreated bacterial infection.

Antibacterial positive control received topical application of 20 mg of 2% fusidic acid cream (Fucidine^®^; Leo Pharma GmbH, Neu-Isenburg, Germany). This corresponds to 0.4 mg of active fusidic acid ingredient (20 mg cream × 2% = 0.4 mg active ingredient), applied evenly across the 9 cm^2^ treatment area. The cream was allowed to remain in contact with the tissue for 8 h under the same Franz cell conditions as the oil treatments. Fusidic acid was tested on skin samples from three randomly selected dogs (*n* = 3) rather than all five donor animals. This decision was based on (1) comprehensive validation by Ehling et al. (2025) [2] and (2) fusidic acid serving solely as a methodological control to verify model responsiveness, not as an experimental treatment under investigation. Resource allocation was prioritized toward the primary research question (ozonated oil efficacy) while maintaining sufficient controls to validate system performance.

Negative controls consisted of non-infected skin samples (no MRSP inoculation) subjected to the same preparation protocol, disinfection, and Franz cell mounting procedures to confirm the absence of contaminating microorganisms and validate the disinfection protocol.

### 2.5. Infection Model and Franz Cell Diffusion Cell Setup

The ex vivo canine dermis model was established using Franz diffusion cells following the validated methodology described by Ehling et al. [2].

After thawing the skin, the hair was shaved and 3 × 3 cm pieces were cut. Samples were rehydrated for 30 min in sterile phosphate-buffered saline (PBS, pH 7.4, 37 °C). The skin was then disinfected by dipping five times (5 s total contact time) into Desmanol^®^ pure disinfectant (Schülke & Mayr GmbH, Norderstedt, Germany) to eliminate commensal bacteria. After disinfection, samples were gently blotted dry with sterile gauze. A Derma stamp (DRS^®^ adjustable 140-pin stamp, 0–3 mm, P-Beauty Oleg Wernik, Biberach, Germany) was used to penetrate the skin, inserting it 300 µm deep. This creates microchannels that facilitate bacterial penetration into the superficial dermis, simulating the compromised barrier function present in clinical pyoderma.

A bacterial suspension was prepared by adjusting MRSP strain 5463/1/22 culture turbidity to 0.5 McFarland standard, which corresponds to approximately 1.5 × 10^8^ CFU/mL.

This suspension was then diluted 1:15 in sterile PBS (pH 7.4) to achieve a final concentration of 1 × 10^7^ CFU/mL. Subsequently, 100 µL of this diluted suspension (containing approximately 1 × 10^6^ CFU total) was applied by micropipette to each skin sample mounted in the Franz cell, distributing the inoculum evenly across the 9 cm^2^ surface. Negative control samples received 100 µL of sterile PBS instead of bacterial suspension.

Prepared skin samples were mounted onto Franz diffusion cells (PermeGear, Inc., Hellertown, PA, USA) with the dermal surface facing upward into the donor chamber and the cut dermal–subcutaneous interface facing downward into the acceptor chamber. The acceptor chamber (volume: 5 mL) was filled with sterile PBS (pH 7.4) and maintained at 38 °C using a circulating water bath to approximate physiological canine body temperature. A magnetic stirrer in the acceptor chamber ensured continuous mixing. The skin was allowed to equilibrate in the Franz cell environment for 30 min prior to bacterial inoculation.

The skin was treated again with the Derma stamp and then stretched over a Franz cell. The acceptor chamber was filled with sterile phosphate-buffered saline (PBS) at 38 °C. The donor chamber was sealed with a plastic cap to maintain humidity and prevent contamination while allowing air exchange. Samples were incubated for 16 h at 38 °C to establish infection.

### 2.6. Topical Treatments

After 16 h of infection, treatments were applied and maintained for 8 h. Oil treatments consisted of 300 µL of either ozonated olive oil or placebo olive oil, applied evenly across the skin surface and gently massaged for uniform distribution (as described in Section 2.4). Fusidic acid controls received 2% fusidic acid cream, applied evenly across the 9 cm^2^ surface using a sterile spatula. All Franz cells were covered with plastic lids to prevent evaporation and maintain humidity while allowing air exchange. The sealed chambers maintained skin hydration and created a semi-occlusive environment approximating clinical dressing conditions. All procedures were performed using aseptic technique in a laminar flow hood. The experimental period was completed after 24 h in total (16 h infection + 8 h treatment).

### 2.7. Sample Processing and Bacterial Quantification

After completion of the 24 h experimental period (16 h infection + 8 h treatment), skin samples were removed from Franz cells and immediately processed. Each skin sample was divided into two portions using a sterile scalpel blade: one for histological examination and the other for bacterial determination.

Histopathological examination was performed by a board-certified pathologist at the Department of Pathology, where the skin was fixed in 10% neutrally buffered formalin, embedded in paraffin and then stained with hematoxylin and eosin (HE) according to a standard protocol [14]. Sections were evaluated qualitatively for the presence, distribution, and localization of bacteria (superficial dermis, perifollicular, deep dermis) based on characteristic cocci morphology and clustering patterns, and the overall tissue integrity and any pathological changes. The comparison between groups was based on qualitative assessment and tissue changes across all stained sections, not on quantitative counting of bacteria per microscopic field. Contaminating organisms would be identifiable by distinct morphology (e.g., Clostridia: large rods).

From the remaining tissue, an 8 mm punch biopsy was obtained for colony counting. The skin sample was placed into a 2 mL microcentrifuge tube containing 500 µL of sterile 0.9% NaCl solution (pH 6.5–7.0) and three 2 mm diameter glass beads. The tissue was mechanically disrupted by vortexing at maximum speed for 30 s, resting for 30 s, and repeating this cycle three times to ensure thorough bacterial extraction from the tissue matrix.

The resulting bacterial suspension was serially diluted in sterile 0.9% NaCl (10^−1^ to 10^−7^ dilutions). From each dilution, 200 µL was plated in duplicate onto selective agar plates (MRSA 2 Brilliance Agar; Oxoid Deutschland GmbH, Wesel, Germany). This chromogenic selective medium was chosen because it reliably detects MRSP while suppressing growth of other bacteria, facilitating enumeration of pure MRSP colonies [15]. Plates were incubated aerobically at 37 °C for 18 h, after which the CFU values were counted. Results are expressed as log_10_ CFU per sample.

**Figure 1:**
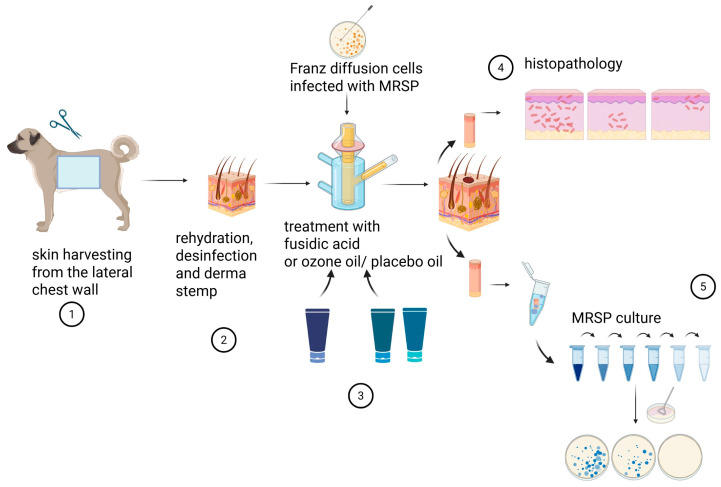
Overview of the experimental processing: 1. Collection of skin sample from lateral chest wall. 2. Shaving of dog skin, rehydration in phosphate-buffered saline (pH 7.4), use of Derma stamp. 3. Skin is placed to Franz cell, infected with MRSP for 16 h and then treated with fusidic acid, ozonated oil or placebo oil for 8 h. 4. After 24 h biopsy is taken for histopathological examination. 5. Additional biopsy is taken for detection of quantitative bacteria (colony-forming units, CFU), using serial dilution with plating on selective agar.

For the study, five different dogs were used. A negative control with fusidic acid was performed on the skin of three randomly chosen dogs. The control without skin infection, meaning only after disinfection to confirm the absence of foreign contamination, was performed with two skin samples (Figure 1).

### 2.8. Statistical Analysis

GraphPad Prism Software (version 10.4.1, GraphPad Software Inc., Boston, MA, USA) was used for analysis. The experimental design and statistical approach followed the validated methodology established by Ehling et al. [2] for this Franz cell model system.

Five dogs contributed skin samples to final analysis, with one initially included dog excluded due to insufficient MRSP colonization following the 24 h experimental period. Each dog contributed one skin sample per treatment condition, with each Franz cell representing one independent experimental unit (no technical replicates per dog per treatment). Final sample sizes were MRSP-only controls (*n* = 5 dogs, 5 Franz cells), MRSP + ozonated oil (*n* = 5 dogs, 5 Franz cells), MRSP + placebo oil (*n* = 5 dogs, 5 Franz cells), MRSP + fusidic acid (*n* = 3 dogs, 3 Franz cells), and negative controls (*n* = 2 dogs, 2 Franz cells). See Table 1.

Bacterial counts were log_10_-transformed for analysis. The Kruskal–Wallis test with Dunn’s multiple comparison post hoc test was applied to compare CFU counts between treatment groups. Dog identity was not treated as a random effect in the statistical model, consistent with the approach validated in Ehling et al. [2]. We acknowledge that inter-individual variability in skin properties (thickness, lipid composition, barrier integrity) was not formally modeled, though the lack of difference between treatment groups was consistent across all donor animals. Statistical significance was defined as *p* < 0.05. Data are presented as median values with 95% confidence intervals.

## 3. Results

### 3.1. Antibacterial Effects In Vitro

**Figure A1:**
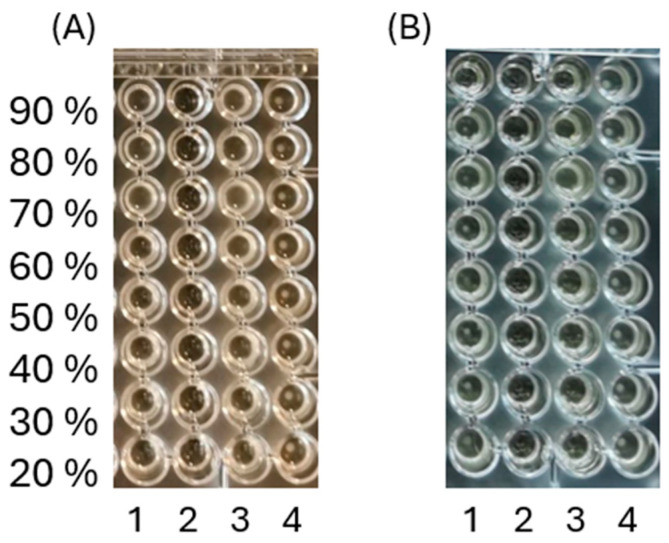
This figure shows part of a round-bottom 96-well plate containing ozone oil at varying concentrations, as well as positive and negative growth controls. The first column contains the oil concentrations with bacterial suspension added, with increasing concentrations from 20% to 90%. The second column shows the negative control, consisting of broth with saline only. The third column is treated with oil and saline, also serving as a negative control. The last column is the positive control, containing Müller–Hinton Broth with bacterial suspension. (**A**) Ozone oil: The MRSP isolate shows button formation only in the positive growth control (4), indicating bacterial growth. No bacterial growth is detected in the negative controls and ozone oil-treated wells; these wells show clear fluid (1 + 2 + 3). (**B**) Placebo oil: The MRSP isolate shows button formation in the placebo oil-treated wells (1), but not at the highest concentration of 90% or in the positive control (4). Both negative controls show clear fluids (2 + 3).

The MIC values were determined for both ozonated oil and placebo oil. The MIC value for ozonated oil was less than 20% (*n* = 5), meaning that bacterial growth was inhibited at the lowest tested concentration of 20% (*v*/*v*), indicating that the true MIC lies at or below this value. For placebo oil, a reduction was detectable up to 90% placebo oil (*n* = 5) (see Appendix A, Figure A1).

The results of the time–kill kinetic analysis, which assessed the rate and extent of bacterial killing over defined contact periods (5, 10, 20, and 60 min), are shown in Appendix A Table A2. The ozonated oil exhibited a bactericidal effect, achieving complete elimination of viable bacteria (0 CFU/mL) by 10–20 min in most replicates, compared to the placebo oil, which maintained high bacterial counts at the same time points. In four out of six experiments, the placebo oil also showed a reduction in viable counts after 60 min of incubation. The minimum bactericidal concentration (MBC) was not formally determined as a separate endpoint.

### 3.2. Histopathological Examination

Histopathological examination of all skin samples was performed as part of the study protocol (Section 2.7). None of the included samples showed pre-existing pathological changes (e.g., inflammatory infiltrates, epidermal hyperplasia, or other signs of dermatitis) beyond the experimentally induced changes.

Histopathological examination revealed adequate tissue preservation across all samples, with most specimens showing good to moderate structural integrity. The epidermis was largely preserved, though segmental epidermal–dermal separation artifacts were observed in several samples, likely attributable to the freeze–thaw process and experimental Derma stamp manipulation.

**Figure 2:**
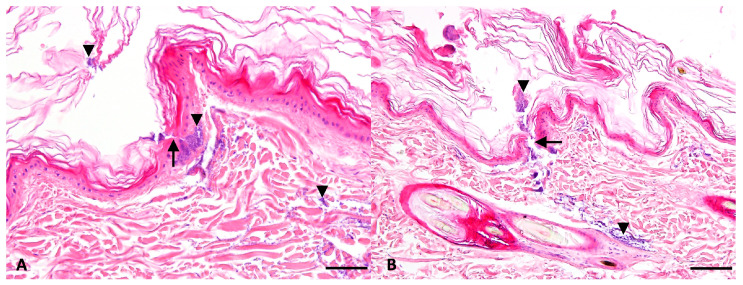
Histopathological examination of MRSP-infected canine skin samples (hematoxylin and eosin staining). (**A**) Bacterial aggregates in the superficial dermis directly beneath the epidermal disruption. MRSP with placebo oil. (**B**) Perifollicular bacterial colonization. MRSP with ozone oil. Arrows indicate epidermal separation caused by the Derma stamp. Arrowheads indicate bacteria (cocci). Scale bars: (**A**) = 50 µm; (**B**) = 100 µm.

Bacterial colonization patterns were consistent with the invasion level in a superficial pyoderma. Cocci were predominantly localized in the superficial dermis across infected samples, with occasional perifollicular distribution. Importantly, no bacteria were detected in hair follicles, and deep dermal or subcutaneous bacterial localization was rare (Figure 2). Semi-quantitative assessment of bacterial density across all infected samples revealed mild to moderate superficial dermal bacteria observed in 15/15 (100%) infected samples (MRSP-only, ozonated oil, and placebo groups combined), mild perifollicular bacteria in 6/15 (40%) samples, and mild deep dermal bacteria in 2/15 (13%) samples. No differences in bacterial distribution patterns were noted between the three infected treatment groups.

No histological differences were observed between MRSP-infected samples treated with ozonated olive oil, placebo oil, or left untreated. Bacterial presence in the superficial dermis was comparable across all infected groups. Notably, fusidic acid-treated samples showed bacteria in histological sections despite negative microbiological cultures.

Based on histopathological examination, no bacteria with morphology inconsistent with staphylococcal cocci (e.g., large rods suggestive of Clostridial contamination) were identified in any of the samples.

### 3.3. Microbial Colonization

**Figure 3:**
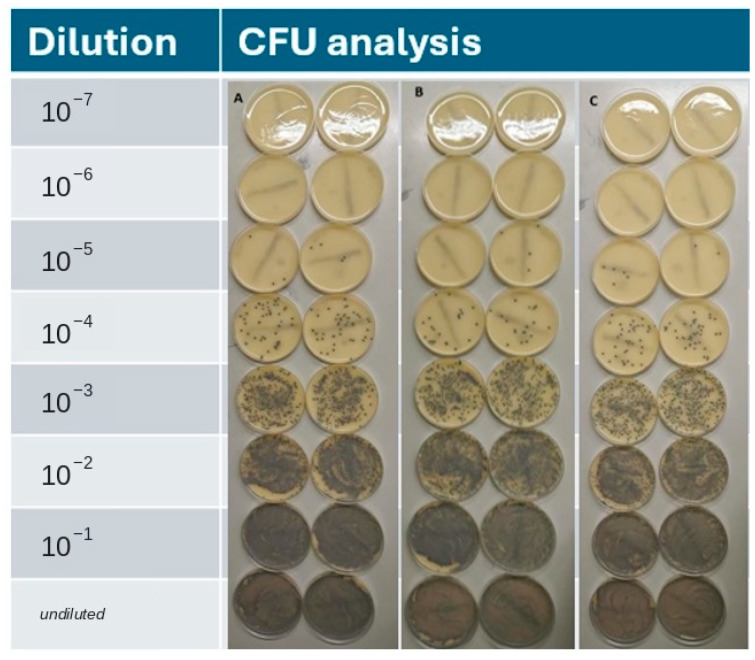
Exemplary analysis of colony-forming units (CFU) after 24 h, blue MRSP colonies on selective agar: (**A**) control, only MRSP, (**B**) MRSP with ozone oil and (**C**) MRSP with placebo oil.

Quantitative bacterial culture revealed that ozonated olive oil treatment failed to reduce MRSP colonization, with no significant difference in CFU counts compared to untreated infected samples (Figure 3).

**Figure 4:**
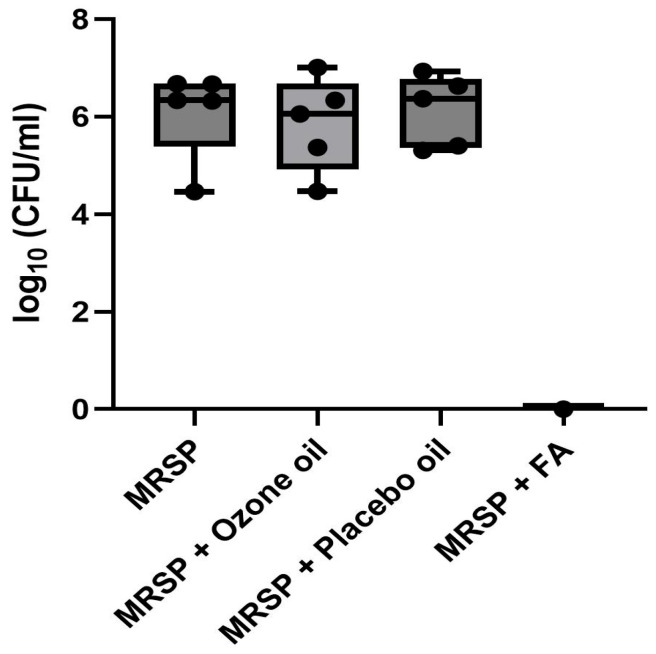
Analysis of CFU after 24 h, number of CFU expressed in log10, *n* = 5 dogs; median with 95% confidence interval. CFU: colony-forming units; FA: fusidic acid. No significance among the three primary treatment groups (Kruskal–Wallis with Dunn’s post hoc test; MRSP vs. MRSP + ozone oil *p* > 0.999; MRSP vs. MRSP + placebo oil *p* > 0.999; MRSP + ozone oil vs. MRSP + placebo oil *p* > 0.999). Formal pairwise statistical comparisons with the fusidic acid group were not performed due to unequal sample sizes (*n* = 5 vs. *n* = 3) and the role of fusidic acid as a methodological control to verify model responsiveness rather than as an experimental treatment. Median and 95% confidence intervals are displayed.

Additionally, the treatments with ozonated oil and placebo control also showed no significant difference. The treatment with fusidic acid showed that there was no growth on the skin samples (Figure 4). 

## 4. Discussion

In this study, topical application of a high-peroxide-value ozonated olive oil blend (800 mEq O_2_/kg) did not reduce MRSP colonization in an ex vivo canine dermis infection model, despite clear in vitro antibacterial activity. Our in vitro susceptibility testing showed that the ozonated olive oil formulation exhibited bactericidal activity against MRSP strain 5463/1/22. The MIC of ozonated oil was less than 20% (*v*/*v*), substantially lower than the 90% (*v*/*v*) concentration required for placebo olive oil, indicating that ozonation provides significant antibacterial enhancement beyond the base oil vehicle. Time–kill kinetic analysis further confirmed ozonation-specific effects: ozonated oil consistently reduced bacterial counts across all six independent experiments, whereas placebo oil showed activity in only four of six replicates and required extended incubation periods (over 60 min) to achieve comparable bacterial reduction. It should be noted that while MIC and time–kill kinetics were determined, the MBC was not formally established as a separate endpoint, which limits the full characterization of the bactericidal versus bacteriostatic activity profile of this ozonated oil formulation. Future studies should include formal MBC determination to characterize the antibacterial properties of ozonated oils more completely.

The divergence between in vitro efficacy and ex vivo failure suggests that the product has intrinsic bactericidal capacity against MRSP when direct contact occurs in aqueous suspension (MIC < 20% *v*/*v*), but under the conditions tested in the ex vivo model (a single 8 h topical application following 16 h bacterial colonization), ozonated olive oil does not provide sufficient antibacterial activity against MRSP to justify its use as a standalone topical therapy for canine pyoderma.

Our experimental design compared ozonated olive oil against multiple controls: (1) placebo olive oil (same vehicle without ozonation), (2) untreated infected controls (MRSP inoculation without any topical application), and (3) fusidic acid (positive antibacterial control). This multi-control approach allowed differentiation between ozonation-specific effects, vehicle-related effects, and baseline infection progression. The lack of significant difference between ozonated oil, placebo oil, and untreated infected controls (all showing similarly high CFU counts, Figure 4) demonstrates that neither the ozonation process nor the olive oil vehicle contributed meaningful antibacterial activity under our experimental conditions.

The ex vivo canine dermis Franz cell model employed in this study was recently validated for MRSP infection research by Ehling et al. [2] using the identical bacterial strain (MRSP 5463/1/22), infection protocol (1 × 10^7^ CFU inoculum, 16 h colonization), treatment duration (8 h), and statistical approach. The concordance between our experimental parameters and those of Ehling et al. [2] enables direct comparison of antibacterial efficacy across different therapeutic agents within a standardized system.

Fusidic acid was tested on skin from three dogs rather than all five donor dogs. This decision was based on two factors: First, Ehling et al. [2] comprehensively validated fusidic acid efficacy in this model system using the identical MRSP strain, infection protocol, and treatment duration, demonstrating consistent complete bacterial eradication across *n* = 5 samples from five dogs [2]. Second, fusidic acid served solely as a methodological positive control to verify model responsiveness to an established antibacterial agent, not as an experimental treatment under investigation. Therefore, we prioritized donor skin allocation toward the primary experimental question (ozonated oil vs. placebo oil efficacy), while including limited fusidic acid controls sufficient to validate system performance. The complete bacterial eradication observed in our fusidic acid control samples (CFU below detection limit) confirmed model responsiveness and validated our experimental system, consistent with Ehling et al.’s findings [2].

Histopathological examination confirmed that our ex vivo inoculation model successfully replicates the invasion level in a superficial pyoderma. Bacteria were predominantly confined to the superficial dermis, with occasional perifollicular distribution at sebaceous gland level, while deep dermal invasion was rare. This distribution mirrors predominantly the clinical presentation of superficial bacterial pyoderma in dogs, where *S. pseudintermedius* colonizes the stratum corneum and superficial dermis without extending into deeper tissue layers [16]. The absence of histological differences between ozonated oil-treated, placebo-treated, and untreated infected samples further supports the quantitative culture findings and confirms that ozonated olive oil did not reduce bacterial burden at the tissue level in this model. The discrepancy between histological and microbiological findings in fusidic acid-treated samples, where bacteria were histologically visible despite negative cultures, reflects a methodological limitation of histopathology in distinguishing viable from non-viable bacteria. Killed bacterial cells retain their morphological appearance in tissue sections and remain detectable by light microscopy. This observation highlights the importance of combining histological assessment with quantitative culture methods when evaluating antibacterial efficacy in tissue-based models.

The results of this ex vivo model stand in contrast to previous in vitro studies demonstrating bactericidal effects of ozonated vegetable oils against staphylococci. Slavinskienė et al. reported dose-dependent antibacterial activity of various ozonated vegetable oils against *S. pseudintermedius* isolates of veterinary origin, with higher peroxide values correlating with greater efficacy [10]. Notably, the ozonated olive oil used in the present study had a peroxide value of 800 mEq O_2_/kg, more than double the highest value tested by Slavinskienė et al. (382 mEq O_2_/kg). However, recent evidence suggests that peroxide value alone does not reliably predict antibacterial efficacy [17]. Dominguez et al. demonstrated that antibacterial activity peaks at intermediate ozonation levels and does not increase further at higher peroxide values [17]. This may reflect differences in the chemical composition of ozonated vegetable oils produced under varying ozonation conditions: prolonged ozonation generates different proportions of primary ozonides, secondary decomposition products (aldehydes, carboxylic acids such as azelaic acid), and lipid peroxides, each with distinct antibacterial potency and tissue penetration characteristics [17].

Silva et al. demonstrated that ozonated vegetable oils effectively disrupted biofilms produced by methicillin-resistant *Staphylococcus aureus* from diabetic foot ulcers [18]. Sechi et al. showed that ozonated sunflower oil was effective against both methicillin-susceptible and methicillin-resistant *S. aureus* and *S. epidermidis* strains, though complete inactivation required 60 min for susceptible strains and up to 180 min for resistant strains [7]. Díaz et al. and Puxeddu et al. demonstrated that ozonation of either olive or sunflower oil results in a similar decrease in oleic and linoleic acids, the formation of 1,2,4-trioxolane rings, and the development of potent, broad-spectrum antibacterial activity, whereas the non-ozonated oils are microbiologically inactive. This shows that olive oil and sunflower oil are closely related in chemical class and function, and that the olive oil used in this study killed MRSP in vitro and did not influence the poor response against it [8,19].

In vitro antibacterial assays evaluate direct contact between antibacterial agents and planktonic bacteria in homogeneous, protein-free environments [20], where ozonides have unimpeded access to bacterial targets. Our ex vivo skin model introduces several barriers absent in conventional testing: three-dimensional tissue architecture creating protective bacterial niches [21], extracellular matrix components that may bind or inactivate reactive species [17], and in living tissue, cutaneous antioxidant enzymes that scavenge reactive oxygen species [21]. Notably, the use of frozen–thawed skin in our study likely rendered antioxidant enzymes non-functional. Despite this presumed inactivation, ozonated oil still failed to demonstrate antibacterial efficacy, suggesting that physical barriers such as tissue architecture and matrix binding, rather than enzymatic neutralization, represent the primary obstacles to efficacy in this model. These findings underscore the limited predictive value of in vitro testing for clinical efficacy, consistent with recent studies showing that antibacterial agents with strong in vitro activity frequently fail in ex vivo skin models [20,21].

Another hypothesis for the lack of efficacy observed in our study is the treatment duration of eight hours. While seemingly adequate, this duration may have been insufficient to achieve meaningful bactericidal effects in the tissue environment. Ozonated vegetable oils release reactive oxygen species gradually through decomposition of ozonides; specifically, the trioxolane structure formed during ozonation decomposes slowly, generating local oxygen, hydrogen peroxide, and lipid oxidation products with antibacterial properties [17]. In vitro kinetic studies have demonstrated that ozonated vegetable oils require extended contact times for bactericidal activity: exposure times of at least 5 min are needed to observe significant bacterial reduction, with complete inactivation often requiring 60 min or more depending on bacterial concentration and resistance profile [7,11]. Nevertheless, for the positive control with fusidic acid, eight hours of treatment time in this ex vivo canine model was more than sufficient to eliminate MRSP.

In clinical practice, topical antibacterials are often applied two or more times daily over extended treatment periods, whereas our single-application protocol may not have allowed sufficient time for the antibacterial mechanisms to take effect. Therefore, our negative findings should be interpreted in that “this formulation, applied once for 8 h to ex vivo skin, did not reduce MRSP colonization”, not as definitive evidence that extended or repeated applications would necessarily fail. Future studies should systematically evaluate (1) extended single-exposure durations (24–72 h) to allow greater cumulative oxidative effects; (2) repeated application protocols that mimic clinical practice; (3) occlusive dressing techniques that may enhance tissue penetration and extend contact time; and (4) combination approaches pairing ozonated oils with other antibacterials.

The bacterial inoculum used in this study was selected to ensure consistent, reproducible infection establishment and to approximate bacterial burdens documented in clinical canine superficial pyoderma. This inoculum matches the validated protocol established by Ehling et al. [2] in this model system. Bäumer et al. demonstrated that experimental canine pyoderma models require 10^5^–10^7^ CFU for reliable infection establishment [16].

However, we acknowledge that high bacterial loads may overwhelm the antibacterial capacity of slowly acting agents like ozonated oils, which release reactive oxygen species gradually through ozonide decomposition [20,21]. This effect may be particularly pronounced given our 8 h treatment duration. Lower inocula (10^3^–10^4^ CFU) might better model early-stage infections where the ratio of antibacterial molecules to bacterial cells is more favorable. Ozonated oils might demonstrate efficacy as preventive or early-intervention agents (applied to minimally contaminated wounds) even if ineffective against established infections with high bacterial burdens. Future dose–response studies evaluating antibacterial activity across bacterial burden ranges (10^3^–10^7^ CFU) would help define appropriate clinical indications.

An additional limitation of this study is the use of a single MRSP isolate (strain 5463/1/22), which restricts the generalizability of our findings across the species. Staphylococcal strains, including MRSP, exhibit substantial genetic and phenotypic diversity that can influence antibacterial susceptibility [22,23]. Different MRSP strains may vary in biofilm formation capacity, surface charge, lipid composition, and metabolic activity, all of which could theoretically affect interactions with oxidative antibacterials like ozonated oils [24]. The use of a single validated strain was justified for initial proof-of-concept evaluation and enabled direct comparison with the extensive validation work by Ehling et al. [2] using this identical isolate. However, definitive conclusions about ozonated oil efficacy against MRSP as a species would require testing against a genetically diverse panel of clinical isolates representing different clonal lineages, resistance profiles, and geographic origins. Future studies should evaluate multiple MRSP strains (minimum *n* = 10 representing major clonal complexes) and, ideally, include methicillin-susceptible *S. pseudintermedius* comparators to determine whether methicillin resistance mechanisms influence susceptibility to oxidative antibacterials. Only multi-isolate validation can establish whether our negative findings represent strain-specific or species-wide inefficacy of this product formulation.

The relatively small sample size of this study (*n* = 5 dogs per primary treatment group) represents a further limitation. With five biological replicates per group, statistical power to detect moderate treatment effects is limited, and the potential influence of inter-individual variability in skin thickness, lipid composition, and barrier integrity cannot be fully captured. The sample size was determined by tissue availability from clinical euthanasia cases and is consistent with the validated methodology established by Ehling et al. [2] using the same model system. Importantly, the lack of treatment effect was consistent across all five donor dogs, with no individual animal showing a trend toward bacterial reduction following ozonated oil treatment, which reduces the likelihood that a genuine effect was missed due to insufficient power. Furthermore, the fusidic acid positive control demonstrated complete bacterial eradication even with *n* = 3, confirming that the model can detect antibacterial activity when present. Nevertheless, future studies should employ larger sample sizes to improve statistical power and enable more robust analytical approaches, including mixed-effect models with dog identity as a random effect, which would more formally account for inter-individual biological variability.

On the other hand, penetration barriers inherent to intact skin tissue likely limited drug delivery to the site of infection. The stratum corneum functions as a remarkably effective barrier, forming a laminate of compressed keratin-filled corneocytes anchored in a lipophilic matrix that limits drugs’ penetration [8]. Although a Derma stamp was used to create microchannels mimicking compromised skin barrier function, the epidermal layers may still have impeded diffusion of the ozonated oil’s active components. In vitro susceptibility testing exposes bacteria directly to antibacterial agents in liquid medium, eliminating the physical barriers that exist in tissue environments. While lipophilic ozonated oil vehicle can penetrate the skin by partitioning into intercellular lipids [25], the hydrophilic reactive oxygen species released upon ozonide decomposition, including hydrogen peroxide, may be rapidly neutralized by cutaneous antioxidant systems before reaching bacteria embedded in deeper tissue layers [26,27].

Olive oil is not biologically inert and theoretically could modulate skin permeability through lipid partitioning into intercellular spaces and influence bacterial adherence by altering surface hydrophobic properties [6,13]. Despite these theoretical concerns, the practical impact appears minimal: the consistently high and statistically indistinguishable bacterial counts across ozonated oil, placebo oil, and untreated infected groups (Figure 4) suggest that neither the olive oil vehicle nor the Franz cell occlusion substantially altered MRSP colonization relative to baseline infection progression. The complete bacterial eradication observed with fusidic acid treatment further confirms that the Franz cell system can detect antibacterial efficacy when genuine activity is present, validating our negative findings with ozonated oil as representing true inefficacy rather than model-related artifacts. Nevertheless, future studies could benefit from additional controls, including (1) non-occluded infected skin samples maintained in open-air conditions to isolate occlusion effects; (2) non-lipid vehicle controls (e.g., aqueous gel or cream base) to definitively separate lipid-specific effects from ozonation effects; and (3) infected skin treated with non-ozonated vegetable oils of different compositions (e.g., sunflower oil, coconut oil) to determine whether our findings are specific to olive oil or generalizable across lipid vehicles.

The ex vivo Franz cell model employed in this study offers several advantages for evaluating topical antibacterials, including the use of authentic canine skin tissue, controlled infection conditions, and the ability to assess both microbiological and histological outcomes. The model’s validity is supported by previous studies [2] and by the complete inactivation of MRSP by fusidic acid, demonstrating that the experimental system can detect effective antibacterial activity when present. However, limitations must be acknowledged. The use of frozen–thawed skin, while practical for standardization, may alter barrier properties and tissue viability compared to fresh tissue. Additionally, the peroxide value of the ozonated oil (800 mEq O_2_/kg) was based on manufacturer certification, and independent laboratory verification was not performed. While this approach reflects the practical evaluation of a commercially available product, it limits our ability to confirm the exact oxidative activity of the formulation at the time of testing, as peroxide values may decrease during storage [17].

Furthermore, the ex vivo system lacks immune cell infiltration and vascular perfusion present in living tissue, which may limit the antibacterial efficacy of ozonated oils. Ozone therapy is known to exert immunomodulatory effects, including activation of neutrophils, stimulation of cytokine release, and enhancement of phagocytic activity [28,29]. In vivo, neutrophils are the first immune cells recruited to sites of bacterial infection and act synergistically with antibacterial agents to eliminate pathogens; they have also been reported to produce ozone as part of their antibody-dependent bacterial killing mechanism [30]. The absence of these immune responses in our ex vivo model prevents such immunostimulatory interactions, potentially explaining why the direct antibacterial activity of ozonated oil alone was insufficient to reduce MRSP colonization.

The findings of this study have important clinical implications, although their interpretation requires caution. Our results demonstrate that the specific ozonated olive oil product tested (800 mEq O_2_/kg) did not exhibit antibacterial efficacy against MRSP under the ex vivo conditions employed. Clinicians should be aware that antibacterial efficacy reported in vitro may not translate directly into clinical effectiveness, and that product-specific differences may exist among commercially available ozonated oils. Until further studies demonstrate consistent efficacy in tissue-based models, ozonated olive oil should not be relied upon as monotherapy for MRSP skin infections, particularly in cases where therapeutic failure carries significant consequences.

Future studies should explore whether modified time of exposure protocols might yield different outcomes. Extended application times and repeated dosing regimens may enhance antibacterial efficacy. Combination approaches pairing ozonated olive oils with other antibacterial agents, as demonstrated by Ehling et al. with bacteriophages and fusidic acid [2], merit investigation. Additionally, ozonated olive oils may prove more beneficial as adjunctive wound-healing agents rather than primary antibacterials, given their reported anti-inflammatory and tissue-regenerative properties [30]. Moreover, comparative evaluation of ozonated oils from different manufacturers, with varying peroxide values, base oil compositions, and ozonation methods, would help determine whether the negative findings observed in this study are specific to this particular product or represent a broader limitation of ozonated olive oils as a class.

## 5. Conclusions

This study evaluated a specific commercial ozonated olive oil product (800 mEq O_2_/kg) against methicillin-resistant *S. pseudintermedius* using complementary in vitro and ex vivo approaches. While in vitro testing confirmed genuine antibacterial activity, with bacteriostatic effects demonstrated by MIC ≤ 20% (*v*/*v*) and bactericidal effects confirmed by consistent time–kill kinetics across six replicates, ex vivo canine skin testing revealed complete failure to reduce MRSP colonization following a single 8 h topical application, with bacterial counts indistinguishable from untreated controls. These findings are specific to the product and conditions tested and cannot be generalized to other ozonated oil formulations, concentrations, or application regimens. Two important limitations of this study must be explicitly acknowledged: the relatively small sample size (*n* = 5 dogs per primary treatment group), which limits statistical power, and the use of a single MRSP isolate (strain 5463/1/22), which restricts generalizability across the phenotypic and genetic diversity of the species.

The results underscore that in vitro antibacterial activity alone does not predict tissue-based efficacy, highlighting the necessity of ex vivo or in vivo validation before clinical application of topical antibacterial candidates.

Future studies should evaluate whether extended exposure durations, repeated application protocols, occlusive dressing techniques, or combination with other antibacterial agents may yield different outcomes. Until product-specific efficacy is demonstrated in tissue-based or clinical models, clinicians should exercise caution in relying on ozonated olive oil as monotherapy for MRSP skin infections and should prioritize agents with demonstrated ex vivo or in vivo efficacy.

## Figures and Tables

**Table 1:** Experimental grouping including sample size and source for each treatment group.

Treatment Group	Dogs (*n*)	Skin Samples/Franz Cells (*n*)	Dog IDs
MRSP-only control (untreated infected)	5	5	Dogs 1–5
MRSP + Ozonated olive oil	5	5	Dogs 1–5
MRSP + Placebo olive oil	5	5	Dogs 1–5
MRSP + Fusidic acid (antibacterial control)	3	3	Dogs 1–3
Negative control (non-infected)	2	2	Dogs 4–5

## Data Availability

The datasets used and/or analyzed during the current study are available from the corresponding author on reasonable request.

## Abbreviations

The following abbreviations are used in this manuscript:

MRSP	Methicillin-Resistant *Staphylococcus pseudintermedius*
CFU	Colony-forming units
MIC	Minimum inhibitory concentration
CLSI	Clinical and Laboratory Standards Institute
PBS	Phosphate-buffered saline
MBC	Minimum bactericidal concentration
HE	Hematoxylin and eosin

## Appendix A

**Table A1:** Antibiotic sensitivity test of *Staphylococcus pseudintermedius* isolate.

Antibacterial Agent	MIC (µg mL^−1^)	MIC Interpretation
Amoxicillin/Clavulanic acid	16/8	R
Ampicillin	32	R
Cefovecin	8	R
Cephalothin	32	R
Chloramphenicol	32	R
Clindamycin	8	R
Doxycycline	2	R
Enrofloxacin	1	I
Erythromycin	8	R
Florfenicol	2	
Gentamicin	4	S
Oxacillin	4	R
Penicillin G	4	R
Pradofloxacin	0.25	S
Tetracycline	8	R
Trimethoprim/Sulfonamide	4/76	R
Fusidic acid	0.125	S

**Table A2:** Kinetic analyses of ozone oil compared to placebo oil: The experiment was performed with six biological replicates. Incubation times (bacterial suspension with ozone oil or placebo oil) were 5, 10, 20, and 60 min, and the CFU was determined after 24 h.

Setup N	Time (min)	Ozone Oil CFU/mL (log_10_)	PLACEBO Oil CFU/mL (log_10_)
1	5	NC	NC
	10	BDL	NC
	20	BDL	NC
	60	BDL	8200 (3.91)
2	5	NC	NC
	10	2700 (3.43)	NC
	20	BDL	NC
	60	BDL	NC
3	5	NC	NC
	10	1500 (3.18)	NC
	20	BDL	NC
	60	BDL	NC
4	5	BDL	NC
	10	BDL	BDL
	20	BDL	NC
	60	BDL	NC
5	5	NC	NC
	10	BDL	NC
	20	BDL	NC
	60	BDL	BDL
6	5	NC	NC
	10	BDL	NC
	20	BDL	NC
	60	BDL	BDL

NC = Non-calculable, which indicates a high number of CFU (more than 300 CFU on the agar plate, i.e., >3.0 × 10^4^ CFU/mL). BDL = below detection limit (0 CFU on agar plate, representing complete bacterial elimination and >99.99% kill from starting inoculum; log_10_ reduction ≥ 7.18).

**Table A3:** Donor signalment.

Dog	Breed	Sex	Age	Reason for Euthanasia
1	Crossbreed	FN	10 year	Cauda equina syndrome
2	German Wirehaired Pointer	ME	6 year	Meningoencephalitis unknown origin
3	Labrador Retriever	ME	6 year	Pericard effusion
4	German Shepherd	FE	10 year	Trauma after car accident
5	Crossbreed	FE	5 year	Herniated disk

FN = female neutered; FE = female entire (intact); ME = male entire (intact).
